# Oxaliplatin and amlodipine combination induced reversible Brugada phenocopy: A case report

**DOI:** 10.1097/MD.0000000000046181

**Published:** 2025-11-28

**Authors:** Wenhui Bian, Haomei Zhang, Mingchen Liu, Xiaofeng Qi, Jie Li

**Affiliations:** a Shandong University of Traditional Chinese Medicine, Jinan, Shandong, China; b Weifang Traditional Chinese Medicine Hospital, Weifang, Shandong, China.

**Keywords:** amlodipine besylate, Brugada phenocopy, cardiotoxicity, drug interaction, mFOLFOX, oxaliplatin

## Abstract

**Rationale:**

Brugada syndrome (BrS) is a rare and life-threatening cardiac electrophysiological disease, characterized by typical electrocardiogram changes and malignant arrhythmia, which is easy to induce sudden cardiac death. The combination of modified FOLFOX (mFOLFOX) chemotherapy regimen and amlodipine is common in clinical practice, but the Brugada phenocopy (BrP) induced by the combination of mFOLFOX and amlodipine is rarely reported. And its arrhythmogenic mechanism is still unclear.

**Patient concerns:**

A 53-year-old man with rectal cancer was treated with amlodipine besylate on a long-term basis. The Brugada type 1 pattern was identified during routine electrocardiogram (ECG) screening before the tenth chemotherapy.

**Diagnoses:**

Drug-induced type 1 Brugada phenotype.

**Interventions:**

The patient declined further testing (e.g., drug challenge testing, SCN5A genetic testing). Under close monitoring, the tenth cycle of mFOLFOX chemotherapy was successfully completed.

**Outcomes:**

No cardiac adverse reactions occurred during chemotherapy. During subsequent cycles of chemotherapy, the electrocardiogram continued to show BrP type 1 but resolved 27 days after the last chemotherapy. No long-term cardiac complications occurred.

**Lessons:**

In this paper, we analyzed the possible mechanism of oxaliplatin combined with amlodipine induced reversible type 1 BrP, so as to provide clinical reference for the safe medication of chemotherapy patients. At the same time, it is suggested that clinicians should pay attention to the risk of synergistic inhibition of ion channels when chemotherapy drugs are combined with antihypertensive drugs, which provides key clinical evidence for reducing the incidence of cardiovascular complications and optimizing multi-drug combination therapy in cancer patients.

## 1. Introduction

BrS is a relatively rare but potentially fatal inherited cardiac ion channel disorder characterized by an electrocardiogram with ST-segment elevation in the right thoracic leads (V1 to V3), which is either “downslope” or “saddle-shaped.” The core feature of the disease is abnormal electrical activity of the heart, which can lead to rapid, disturbed rhythms of the ventricles (polymorphic ventricular tachycardia or ventricular fibrillation), severely interfering with the heart’s ability to pump blood efficiently, leading to fainting, cardiac arrest, and even sudden death, especially at rest or during sleep.

mFOLFOX is a common clinical chemotherapeutic regimen that consists of 5-fluorouracil, calcium folinate, and oxaliplatin. In recent years, with the widespread use of chemotherapeutic agents in tumor treatment, the risk of cardiovascular complications in patients has gradually increased, and there has been a growing concern about the cardiotoxicity of chemotherapeutic agents (especially oxaliplatin), which may interfere with myocardial electrical activity through inhibition of sodium channels (I_Na_). Amlodipine besylate, as an L-type calcium channel (ICa-L) blocker, may further exacerbate the ionic current imbalance. In this article, we discuss the potential mechanism of type 1 BrP that may be induced by the combination of mFOLFOX chemotherapy and amlodipine besylate and analyze possible drug-drug interactions through a clinical case of a patient with rectal cancer.

## 2. Case report

**Figure 1.**
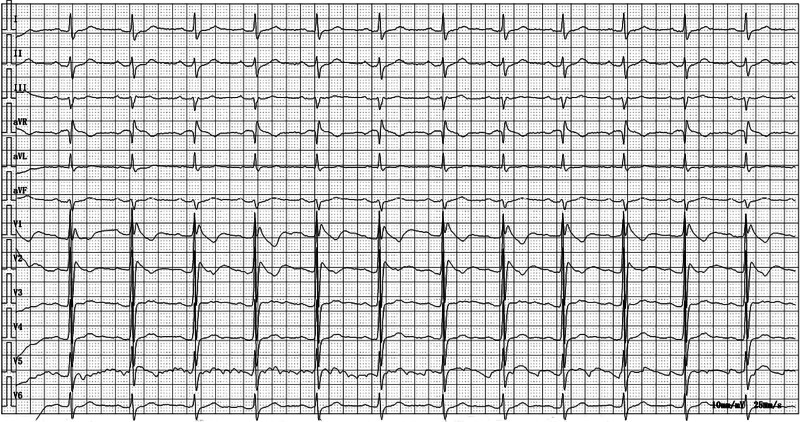
Electrocardiogram obtained on the day of admission for the 10th chemotherapy session.

A 53-year-old man, more than 5 months after surgery for rectal cancer, presented for his 10th mFOLFOX chemotherapy. He underwent preoperative laparoscopic radical rectal cancer surgery + ileostomy, and began to receive a total of 12 cycles of mFOLFOX chemotherapy (oxaliplatin 150 mg, fluorouracil 0.6g i.v. + 4.2g continuous pumping for 46 hours, and calcium folinate 600 mg) regularly after surgery. The patient complained of mild abdominal distension, no abdominal pain, no chest tightness or breath-holding, and denied fever, syncope, palpitations, and nocturnal dyspnea. On admission, blood pressure was measured at 129/85mmHg and heart rate 75 beats/min. ECG showed a new pattern of right bundle branch block, with downward oblique ST-segment elevation and T wave inversion in V1 to V2 leads, consistent with type 1 BrP (Fig. 1). In addition, the patient denied personal and family history of sudden cardiac death and was on regular long-term oral amlodipine 5 mg QD. Laboratory investigations revealed leukocytes of 2.77×10^9^/L, erythrocytes of 4.21×10^12^/L, and hemoglobin of 130 g/L. Serum electrolyte test results showed potassium of 3.82 mmol/L, sodium of 143.2 mmol/L, and chloride of 108.7 mmol/L. Liver and renal functions were normal. Computed tomography of the chest, whole abdomen and pelvis showed postoperative changes in the rectum; Solid micronodules in bilateral upper lobes. Abnormal density below the navel.

**Figure 2.**
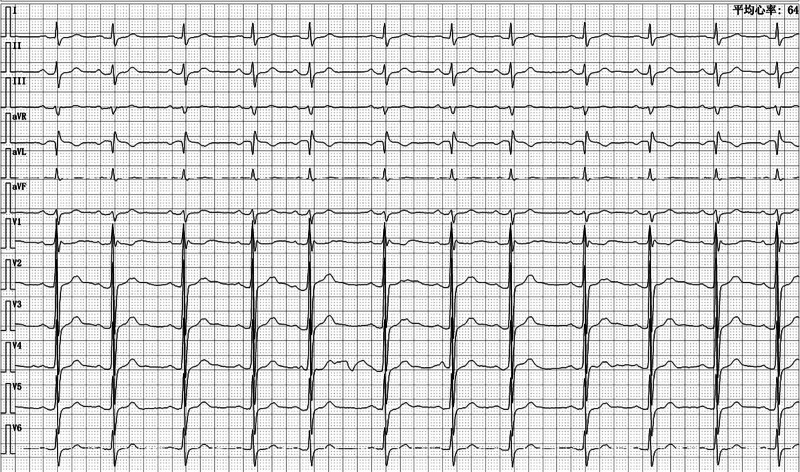
Electrocardiogram rechecked 27 d after the last chemotherapy.

In response to the patient’s new ECG abnormality, further refinement of the examination showed normal myocardial markers, and cardiac ultrasound showed no obvious abnormality of intracardiac structures. After consultation with the cardiology department and other related departments, an electrophysiologic examination for a drug provocation test or SCN5A gene test was recommended to identify BrS, which the patient refused. After being informed of the patient’s condition in detail, the patient decided to continue chemotherapy in a monitored setting. The patient did not experience any discomfort other than bloating during the 10th round of mFOLFOX treatment. At the postdischarge telephone follow-up, the patient was in good physical condition, with no palpitations, syncope, or nocturnal convulsions. The patient received the next round of chemotherapy every 14 days, during which the ECG continued to show a type 1 Brugada pattern. Twenty-seven days after the final round of chemotherapy, a repeat ECG showed sinus arrhythmia and disappearance of the type 1 Brugada pattern (Fig. 2).

## 3. Discussion

BrS is an autosomal dominant channelopathy associated with mutations in voltage-gated ion channels in cardiomyocytes.^[1]^ Its diagnosis requires the fulfillment of 2 conditions: first, the display of type 1 ECG changes in at least 1 right chest lead (V1 to V2); and second, the clinical presence of ventricular tachycardia, ventricular fibrillation, syncope, sudden cardiac death, nocturnal near-death breathing, palpitations, and chest discomfort. The ST-segment changes in chest leads V1 to V3 can be divided into 3 types: Type 1 shows J-point elevation (≥2 mm), ST-segment downward-sloping elevation, symmetrical inverted T waves, and ST-T changes of dome type; Type 2 shows J-point elevation (≥ 2 mm), ST-segment saddle-shaped elevation, positive or low-flat T waves, and ST-T changes of saddle type; and Type 3 shows ST-segment elevation <2mm and “dome-shaped” or “saddle-shaped” ST-T changes. Only type 1 is clinically significant for the diagnosis of BrS, while types 2 and 3 usually require further evaluation. In patients without clear symptoms, medical history, or family history of BrS, the presence of type 2 or type 3 Brugada-like changes on the ECG in response to a triggering factor or pathological condition is clinically referred to as a BrP.^[2]^ The common clinical triggers or etiologies of BrP include metabolic abnormalities (potassium abnormalities are the most common), pulmonary embolism, mechanical compression, myocardial and pericardial diseases, rhabdomyolysis, acute intracranial hemorrhage, and drug toxicity, which are highly reversible.

As a first-line treatment option for colorectal cancer, mFOLFOX is suitable for adjuvant treatment after surgery and palliative treatment for advanced cancer, and is widely used clinically. Cardiovascular involvement, such as arrhythmia and cardiac insufficiency, has been reported, but the occurrence of type 1 BrP during the application is rare, and its pathophysiologic mechanism is still unclear. This study proposes a potential mechanism by which mFOLFOX may induce BrP in the context of the case.

The mFOLFOX regimen leads to type 1 BrP, possibly as a result of oxaliplatin interacting with amlodipine benzenesulfonate, which the patient has been taking orally for a long period of time, by blocking sodium channels. This process can be further explained by the concept of “repolarization reserve”: in normal myocardium, functional abnormalities of a single ion channel can maintain repolarization homeostasis through compensatory activation of other channels;there is a “repolarization reserve.”^[3]^ In the present case, we cannot definitively assert that the combination of the mFOLFOX regimen (containing oxaliplatin) and amlodipine besylate is the sole causative agent of BrP, but the synergistic effect of the 2 in combination with their characteristic actions on myocardial ion channels is most likely the central driver, i.e., oxaliplatin inhibits the peak of sodium currents and delays sodium channel inactivation,^[4]^ and amlodipine besylate specifically blocks L-type calcium currents^[5]^ When this injury exceeds myocardial compensatory capacity, the repolarization reserve is depleted, leading to a relative predominance of outward currents (especially Ito) and an increase in right ventricular epicardial and endocardial repolarization dispersion, which ultimately reveals the characteristics of type 1 BrP.

The essence of BrP is an imbalance of myocardial ionic currents, and the typical abnormalities of BrS arise from genetic defects in sodium channels (e.g., mutations in the SCN5A gene).^[6]^ The patient in this case did not have a relevant family history or previous record of cardiac electrophysiologic abnormalities, and therefore preferred drug-association-induced acquired alterations, i.e., disruption of ion channel function by exogenous drug action, rather than a congenital genetic defect. This is consistent with the repolarization reserve theory that “drug-induced ion channel abnormalities can be reversed after removal of the causative agent,” which is further supported by the disappearance of the ECG abnormality 27 days after the patient’s final chemotherapy in this case.

In the case of BrP induced by the combination of mFOLFOX and amlodipine benzenesulfonate, care must be taken to differentiate it from a true BrS syndrome. Although the core mechanisms of the 2 are similar (both involving abnormalities in sodium and calcium currents), the underlying pathologic basis and clinical significance may be different: hereditary BrS is a permanent channel defect, whereas the drug-induced electrophysiologic alterations in this case are reversible, and the key difference is the presence of a congenital deficit in repolarization reserve. The synergistic effect of oxaliplatin and amlodipine besylate is not a direct trigger of channel mutations, but rather depletion of repolarization reserve by superimposed inhibition of inward current.

Although the lack of genetic testing or detailed electrophysiologic findings in this case makes it difficult to completely rule out an underlying genetic predisposition in the patient, the combination of features such as “no family history of sudden death” and “abnormal reversal after drug discontinuation” suggests a significantly higher likelihood of drug-combination triggering. Nevertheless, given the patient’s typical type 1 BrP during chemotherapy, it is still necessary to be alert to her potential arrhythmic risk, and clinical management needs to balance oncological treatment and cardiac safety. Based on the principle of protection of repolarization reserve, there is no specific intervention for this kind of drug-induced electrophysiological abnormality, and it is recommended that close monitoring is the core: regular review of electrocardiograms (focusing on leads V1 to V2) and electrolytes (e.g., hypokalemia may further reduce the repolarization reserve), avoiding combining with other drugs affecting the ion channels; assessing the risk in time when abnormalities occur, and adjusting chemotherapy regimens or replacing antihypertensive medications if necessary.

This study is a single-case report with limited sample size, and the conclusions need to be further verified by multi-center and large-sample studies. Second, because the patient refused to undergo SCN5A genetic testing and electrophysiological drug provocation tests, a potential congenital ion channel gene defect could not be completely ruled out in the patient. Third, the mechanism analysis of BrP induced by oxaliplatin and amlodipine besylate is only based on literature and theoretical derivation, and lacks the support of direct experimental data from patients.

## 4. Conclusions

The combination of oxaliplatin and amlodipine besylate may deplete myocardial repolarization reserve through dual inhibition of sodium and calcium currents, disrupting ionic balance and inducing BrP, which not only reveals the potential risk of ECG abnormalities associated with mFOLFOX regimen, but also provides a new perspective on the mechanism and management of drug-induced BrP, and also suggests that the clinic needs to pay attention to cardiovascular risk of polypharmacy in oncology patients during chemotherapy. By combining genetic screening, drug management, and electrophysiological monitoring, we can provide a more personalized treatment strategy for such patients and reduce the risk of drug-induced ECG abnormalities.

## Author contributions

**Conceptualization:** Wenhui Bian, Haomei Zhang.

**Investigation:** Wenhui Bian, Haomei Zhang, Xiaofeng Qi.

**Methodology:** Mingchen Liu.

**Writing – original draft:** Wenhui Bian.

**Writing – review & editing:** Jie Li.
